# Influence of Cellulose Nanoparticles on Rheological Behavior of Oil Well Cement-Water Slurries

**DOI:** 10.3390/ma12020291

**Published:** 2019-01-17

**Authors:** Zhengjie Tang, Runzhou Huang, Changtong Mei, Xiuxuan Sun, Dingguo Zhou, Xiuqiang Zhang, Qinglin Wu

**Affiliations:** 1 College of Materials Science and Engineering, Nanjing Forestry University, Nanjing 210037, China; zhengjietang@163.com (Z.T.); mei@njfu.edu.cn (C.M.); dgzhou1111@163.com (D.Z.); 2 School of Renewable Natural Resources, Louisiana State University AgCenter, Baton Rouge, LA 70803, USA; xsun17@lsu.edu; 3 College of Materials Science and Engineering, Southwest Forestry University, Kunming 650224, China; 4 Key Biomass Energy Laboratory of Henan Province, Zhengzhou 450008, China; Zhangxiuqiang@163.com

**Keywords:** cellulose nanoparticles, oil well cement, rheology, rotational viscometer

## Abstract

Performance of hardened oil well cement (OWC) is largely determined by the rheological properties of the cement slurries. This work was carried out to investigate the effect of water- to-cement ratio (WCR) and cellulose nanoparticles (CNPs), including cellulose nanofibers (CNFs) and cellulose nanocrystals (CNCs), on rheology performance of OWC-based slurries using a Couette rotational viscometer coupled with rheological models. The yield stress and viscosity of neat OWC slurries had a decreasing trend with the increase of WCRs. The suspension became increased unstable with the increase of WCRs. The properties of CNPs, including rheological behaviors, surface properties and morphology, determine the rheological performance of CNP-OWC slurries. In comparison with CNC-OWC slurries, the gel strength, yield stress and viscosity of CNF-OWC slurries were higher as CNFs were more likely to form an entangled network. The gel strength, yield stress and viscosity of CNP-OWC slurries increased with reduced CNF size through regrinding and the proportion of CNFs in the mixture of CNFs and CNCs, respectively.

## 1. Introduction

Oil well cementing operations play a vital role in the petroleum industry. Oil well cement (OWC) is mainly constituted by Portland cement and other additives including accelerators, retarders, extenders, heavy weight agents, fluid loss additives, lost circulation additives, expansion additives, dispersants and antifoam agents [1,2]. The cementing operation is to form a ring-shaped space between the oil well casing and the structure surrounding the oil well bore, to support and protect the casing, to isolate abandoned sections of well and to cut out the lost circulation zones [3]. For the special case of the oil industry, OWC slurries are sometimes pumped down to in excess of several thousand meters into the ground and face additional challenges compared to ordinary cementing work on the surface. The rheological properties of OWC slurries determine the character of the hardened OWC-based material and help forecast its final mechanical properties and performance during and after processing [1]. Therefore, accurately characterizing the rheology of OWC slurries is critical. The rheological properties of OWC slurries are influenced by multiple factors, such as water-to-cement ratio (WRC), size and shape of cement particle, type and quantity of additives [1].

Because of the individual characteristics including a high surface area to volume ratio and nanoscale effect, nanoparticles have been paid increasing attention as additives in cementitious materials [4]. Cellulose nanoparticles (CNPs), mainly consisting of cellulose nanofibers (CNFs) and cellulose nanocrystals (CNCs), are extracted from various cellulose resources, such as wood, vegetable fiber, chitin and other cellulose resources by mechanical and hydrolysis treatment [5]. CNPs have been used to reinforce cementitious matrix materials. Due to a high surface area to volume ratio, CNPs have the potential to increase chemical reactions of cementitious materials, which neither macrosized plant fibers nor molecular cellulose can do [6]. CNPs show great advantages when being added to cementitious composites as a reinforcing agent with the unique characteristics, such as high Young’s modulus, and large aspect ratio [7]. For cements, CNP additions can alter mechanical properties (such as stiffness and strength), setting times, and rheology [8]. The hydration, transport behavior and microstructure of CNC-cement composites were studied by Flores et al [9]. The results showed that the early stage hydration was delayed, but the later stage hydration was improved. According to the research of Cao et al [10,11], the dispersion was the key to a successful application of CNCs in CNC-cement composites. They also pointed out that sonication treatment for the CNC-rich regions of CNC-cement composites had higher elastic modulus. Mònica Ardanuy et al. [12] investigated the use of CNFs made from sisal by mechanical treatment in the cementitious composites. The results showed that the flexural strength of CNF-cement composites increased 40% and nearly twice compared with cement composites enhanced by sisal fiber, but the fracture toughness was half of sisal fiber-cement composites. Onuaguluchi et al [13] observed that CNFs manufactured from bleached softwood pulp delayed the setting time and increased the Degree of hydration(DOH) of cement with CNF loading of 0.05%, 0.1%, 0.2% and 0.4% of cement weight. The mechanical, thermal, and microstructural of CNF-cement composites were studied by Mejdoub et al [14], in which CNFs were produced by homogenization from eucalyptus pulp. The results showed that most measured properties (i.e., mechanical, thermal, and microstructural) were significantly improved. 

Although OWC-based materials can get much benefit from using CNPs as a modification, the related work in this research area seems very scarce. Until to date, there were only several publications relative to this topic. Sun et al [15] showed that the flexural strength of CNF-OWC composites was increased by 20.7% when CNF/OWC ratio reached up to 0.04 wt.% and the yield stress of CNF-OWC composites increased as the amount of CNFs added increased. The coupled effect of CNFs and graphene nano-platelets was studied with the aid of four different rheological models. The results showed the yield stress was increased and the Vom Berg model was the most consistent with the slurry rheology [16].

The rheological properties of cement are mainly described by shear stress or viscosity as a function of the shear rate using a rheometer like coaxial cylinder viscometer, pile-flow viscometer and vane rheometer [16]. The equipment used to quantify the rheological properties of cement-based materials has a number of limitations, such as being relatively valuable, complicated to operate, harsh to the operating environment, and potentially unsuitable for use in field sites. Many mathematical models have been used to evaluate the rheological properties of a series of cement-based materials [17,18]. The popular rheological models can be divided into two-parameter models and three- parameter models. The Bingham Plastic Model (BPM), Power Law Model (PLM), Casson Model (CM) and Eyring Model (EM) are two-parameter models. The BPM is the most commonly used when the relationship between shear rate and shear stress is linear. The PLM overcomes the drawback of BMP, which can fit the rheological data more adequately under certain nonlinear conditions. However, the PLM does not have the yield stress that can be calculated by BPM. The Modified Bingham Model (MBM), Hershcel-Bulkley Model (HBM), Vom Berg Model (VBM), Vocadlo Model (VM) and Roberston-Stiff Model (RSM) are three-parameter models and HBM combines the advantages of BPM and PLM. Tattersall and Banfill used the BPM to describe the rheological properties of cement slurries at low shear rates with some success [19]. Jones and Talyor used the HBM to fit rheological data obtained by a cone and plate viscometer to exhibit the reversible behavior of cement slurry [20].

The main goals of this study were (1) to evaluate the rheological properties of OWC using a coaxial cylinder viscometer, which is widely used in research and production of oil industry; (2) to study the effect of WCRs on the rheological properties of OWC, and (3) to investigate the effect of CNP additions on the rheological properties of OWC, including the CNP amount, the size of CNFs and the combined effect of CNCs and CNFs.

## 2. Materials and Methods

### 2.1. Materials and Preparation

CNCs (concentration of 2.6 wt.%) from (Alberta Innovates Technology Futures, AITF, Edmonton, Alberta, Canada) and CNFs (concentration of 3.6 wt.%) from University of Maine (UM, Orono, Maine, USA) were used for the study. Class H well cement was provided by Halliburton Energy Services (Houston, TX, USA), ASTM Type II deionized water was provided by VWR International LLC (Radnor, PA, USA).

CNFs were dispersed in water to form 1 wt.% suspension using a magnetic stirrer (IKA RET Basic, IKA-Works, Inc., Wilmington, NC, USA) for 15 min. The suspension was passed 10 and 30 times through a Supermass Collider machine (MKCA6-3, Masuko Sangyo Co., ltd, Tokyo, Japan) at 1500 rpm. The processed suspension was passed through a microfluidics homogenizer (Microfluidizer Processor M-110P, Microfluidics Corp., Newton, MA, USA) five times with operating pressure of 200 MPa.

### 2.2. Characterization of CNPs

Solid films of CNCs and CNFs were obtained by drying CNC and CNF suspensions in a vacuum oven (DZF60620, MTI Corp, Richmond, CA, USA) for several days at a temperature of 50 °C. A Fourier transform infrared spectroscopy (FTIR) (Tensor-27, Bruker Optics Inc.,, Billerica, MA, USA) and X-ray diffractometer (XRD) (Bruker Siemens D5000, Bruker Corp., Billerica, MA, USA) were used to study the chemical and crystalline structure of CNC and CNF films. The FTIR spectra were collected by using a Bruker FTIR analyzer (Tensor-27, Bruker Optics Inc., Billerica, MA, USA) in the transmittance mode. The wavenumber increased from 500 to 4000 cm^−1^ with a step of 4 cm^−1^ by a Zn/Se total reflectance crystal accessory. The crystallinity properties were obtained using an X-ray diffractometer (D5000, Bruker Corp., Billerica, MA, USA) with a supply of Cu-K*α* radiation (*λ* = 0.154 nm) in the angular range of 5° to 60° at a step size of 0.026° (accelerating voltage = 40 kV and current = 30 mA). The degree of crystallinity (*DOC*, %) for each sample was calculated by $DOC\left(\%\right)=100\times \frac{(I_{Max}-I_{Am)}}{I_{Max}}$, where *I_Max_* is the maximum intensity of the principal peak, and *I_Am_* is the intensity of diffraction attributed to amorphous cellulose [17]. Zeta-potential values of CNCs (0.1 wt.%) and CNFs (0.1 wt.%) suspensions were measured by a ZetaTrac analyzer (MicroTrac Inc., Largo, FL, USA). The morphology of nanocomposites was determined by atomic force microscopy (AFM, Bruker Nanoscope VIII Multi-Mode, Billerica, MA, USA). A tapping mode was utilized for morphological observation. Samples were first dispersed in N,N-dimethylformamide solvent. The homogeneous suspensions were then spin-coated on the mica surface, and then dried at the ambient temperature. The samples were subsequently scanned through the variation of cantilever. The parameters for the tip radius and spring constant were 2 nm and 20-80 N/m, respectively. The morphology of ground CNFs was examined using a transmission electron microscope (TEM, JEM 1400, JEOL, Peabody, Massachusetts, USA) at an accelerating voltage of 120 kV. A droplet of CNFs suspension (0.02 wt.%) was placed on the copper grid, which was treated with glow discharge followed by stained using uranyl acetate.

### 2.3. CNP-OWC Sample Preparation

Table 1 shows a summary of the formulations of the cement pastes studied in this work. The WCRs of OWC slurries without CNPs were chosen as 0.40, 0.45, 0.50, 0.55 and 0.60 as control formulations. The CNP-OWC slurries were made using cement, water and CNPs with WCR of 0.5, and the added amount of CNCs and CNFs was 0.05 wt.%, 0.10 wt.%, 0.15 wt.% and 0.20 wt.%, respectively (based on the mass of dry OWC). The CNP-OWC slurries with blended CNCs and CNFs were made using cement, water and CNPs with WCR of 0.5 and the added amount of blended CNPs was 0.15 wt.% (based on the mass of dry OWC). The CNF-OWC slurries with CNFs of different grinding conditions (i.e., 0, 10 and 30 times) were made up of cement, water and CNFs, and the added amount of the CNFs was 0.15 wt.% (based on the mass of dry OWC) at WCR of 0.5.

In preparing the slurries, CNPs were first added into water and stirred by a magnetic stirrer for 15 min at speed of 800 rpm to form a stable suspension, and then target amount of OWC was added to the suspension. The composite slurries were mixed at a rate of 10000 rpm for 1 min.

### 2.4. Rheological Measurement and Data Processing and Rheological Modeling

The rheology of OWC and CNP-OWC slurries was measured by a viscometer (model 35A, Fann Instrument Company, Houston, Texas, USA). The prepared OWC and CNP-OWC samples were homogenized for 10 min with a rotational rate of 150 rpm in an atmospheric consistometer at room temperature. Then the composite slurries were poured into the test cylinder. The shear stress at each rotational speed of viscometer (600, 300, 200, 100, 6 and 3 rpm) (1021, 511, 340, 171, 10 and 5.11 s^−1^, respectively) was recorded. The operating steps of initial and final gel strength include (1) stirring the sample thoroughly at 600 rpm for 1 min, (2) turning off the rotor, setting the gear shift knob to the 3 rpm position and waiting for 10 s, (3) turning on the rotor and recording the maximum torque value (G_i_-initial gel strength, Pa), (4) turning off the rotor and waiting for 10 min, and (5) turning on the rotor and recording the maximum torque value (G_f_-final gel strength, Pa). The operations above were conducted at room temperature. 

Measured shear stress versus shear rate data were modelled using the four rheological models shown in Table 2. The chosen models include one linear model (BPM), and three nonlinear models (MBM, PLM, and HBM). Nonlinear regression analysis was done using Origin software to establish each model parameter for a given model. 

## 3. Results and Discussion

### 3.1. CNP Properties

**Figure 1:**
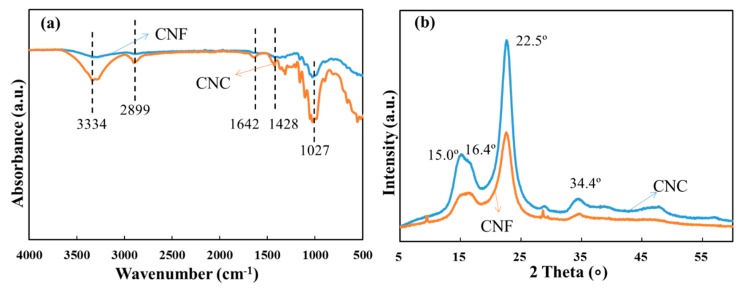
Characterization of CNFs and CNCs: (**a**) FT-IR transmittance spectra; (**b**) X-ray diffraction spectra.

Certain differences between the FTIR spectra of CNCs and CNFs can be seen from Figure 1a. The broad bands in the 3500–3000 cm^−1^ were attributed to O-H stretching vibrations and the peaks at 2899 cm^−1^ were attributed to C-H stretching vibrations. The signal scanned at around 1027 cm^−1^ was due to the C-O stretching vibrations and 1428 cm^−1^ arose from C-H asymmetric deformations. The peak intensities of CNCs were stronger than that of CNFs. The peak intensity of CNCs at 1642 cm^−1^ was stronger than that of CNFs, because of the absorbed water in the non-crystalline region [21]. Figure 1b exhibits the wide-angle XRD spectra of CNCs and CNFs, which showed a typical crystalline structure of cellulose I, with main peaks located at 15.0°, 16.4°, 22.5° and 34.4° corresponding to the crystalline planes of (1$\bar{1}$0), (110), (200) and (004), respectively [22]. The degree of crystallinity (DOC) of CNCs and CNFs are 72.7 and 65.0%, respectively, which are in agreement with the results from previous research [22]. Owing to the manufacturing process that removes the amorphous regions efficiently, the CNCs had a higher crystallinity compared with that of CNFs, which is consistent with the result of FTIR data.

**Figure 2:**
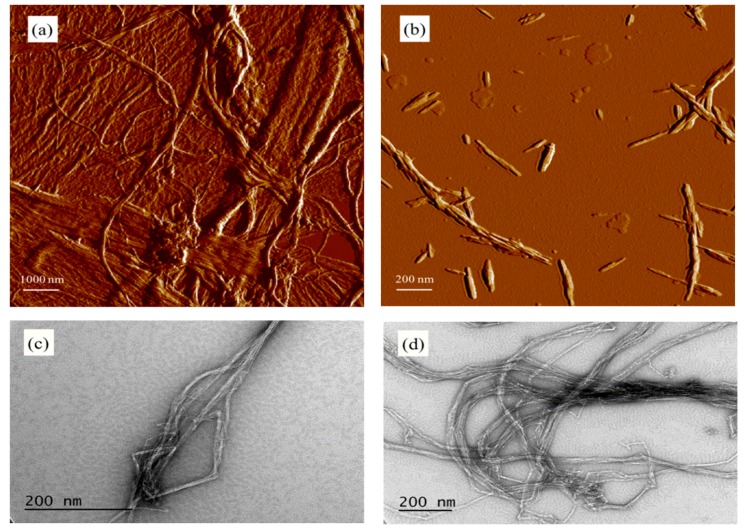
Morphology of CNFs and CNCs, (**a**) and (**b**): Atomic Force Microscopy (AFM) micrographs of CNFs and CNCs; (**c**) and (**d**): TEM micrographs of CNFs with two different regrinding conditions (10 and 30 times), respectively.

The Zeta-potential values of CNCs and CNFs were −39.60 and −15.93 mV, respectively. CNFs showed a highly entangled network composed of microfibril bundles and individual microfibrils, while CNCs were whisker shaped or needle-like particles as shown in Figure 2a,b. Re-grinding of CNFs helped reduce their size. Large bundles existed in raw CNFs (Figure 2a,c) and reground material had a reduced number of large CNF bundles (Figure 2d). The CNFs tended to aggregate more quickly and form network structure when being incorporated with OWC slurry than CNCs, resulting in the difference of rheological properties between CNF-OWC and CNC-OWC slurries as discussed in the later sections.

### 3.2. Effect of WCR on Rheological Properties of OWC Slurries

**Figure 3:**
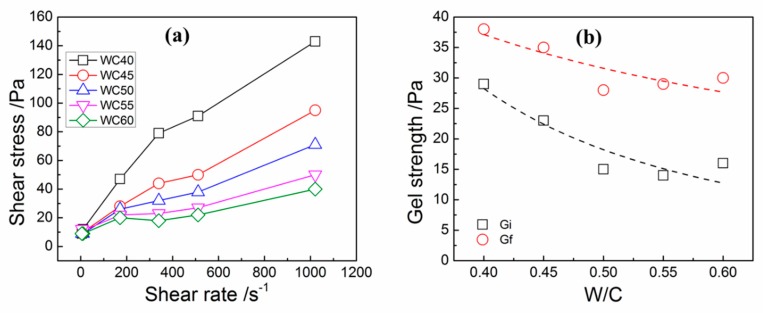
Plots of shear stress versus shear rate for neat OWC slurries (**a**) and their gel strengths (**b**) at different WCRs.

In order to examine the effects of WCRs on the rheological properties of OWC slurries, the neat OWC slurries were formed without any additives. The rheological performance of neat OWC slurries is displayed in Figure 3. Figure 3a reveals that OWC slurries with different WCRs showed significantly different rheological properties. Shear stress increased with the increase of WCRs (WCRs equal to 0.40, 0.45, 0.50 and 0.55) and increased with the increase of the shear rate. When the WCR was 0.60 and the shear rate increased from 10 s^−1^ to 511 s^−1^, the shear stress decreased first and then increased with the increase of the shear rate. Figure 3b illustrates that as the WCR increased, the initial (G_i_, Pa) and final (G_f_, Pa) gel strength showed a decreasing trend. 

Table 3 presents the calculated model parameters of OWC slurries at different WCRs using the chosen four rheology models. As indicated in Table 3, the correlation coefficient, R^2^, values of all predicting models decreased with the increase of WCRs. When the WCRs were 0.55 and 0.60, the HBM cannot be used for data fitting because of increased fluid instability. It has been reported that the HBM is sufficient to depict the rheological properties of neat cement slurries with WCR of 0.30 to 0.40 [23]. Atzeni et al. also used the HBM successfully to fit the rheological data acquired by cone and plate rheometer with WCR of 0.30 to 0.45 [24].

The yield stress of OWC slurry is a crucial property affecting flow behavior, such as flow rate and filling capacity. The stress represents the minimum external force required for a material begins to flow. The yield stress is determined using the shear stress-shear rate data by a given analytical rheological model when the shear rate is zero. It should be noted that yield stress values predicted by BPM and MBM decreased with the increased WCRs, and yield stress values predicted by HBM showed an opposite trend. 

The OWC slurries are more like solid with increased of WCR and harder to start flowing. There are two models including BPM and MBM that contain the parameter of plastic viscosity. It can be observed that the plastic viscosity calculated as listed in Table 3 decreased with the increased WCRs.

In addition to participating in a series of complex chemical reactions, the water in the cement paste also affects the rheological properties of the cement slurry. A series of complex physical and chemical reactions took place immediately when OWC was combined with water. The hydration reaction of OWC consumed some water, but the WCR used in the test far exceeded the amount of water required for the chemical reaction of the OWC [25]. Excessive water can enhance the fluidity and workability of the cement slurry, but increases the instability of the cement slurry at the same time [25]. With the increase of WCR, the increased free water in the cement slurry, which did not participate in the chemical reaction, made the fluidity of the cement slurry better, and thus the plastic viscosity and yield stress of the cement slurry were reduced, and the gel strengths were also reduced. The results are consistent with the study by Gandelamn [8]. The increased instability of the OWC slurries can explain the variability of R^2^ values of the models and the gel strengths including initial gel strength G_i_ and final gel strength G_f_ with the WCR of 0.60 were higher than these with the WCR of 0.50.

### 3.3. Effect of CNPs on Rheological Properties of CNP-OWC Slurries

**Figure 4:**
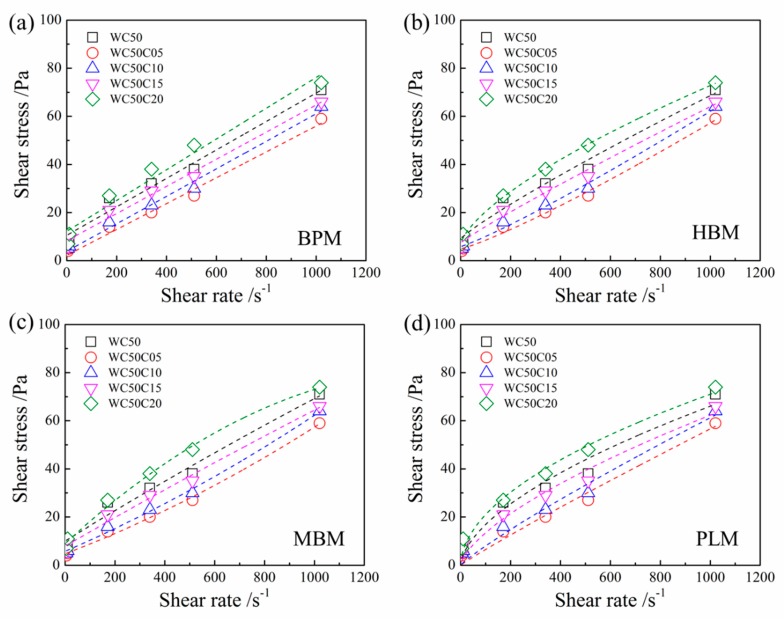
Experimental data and fitted plot of shear stress versus shear rate for CNC-OWC slurries: (**a**) Bingham Plastic Model; (**b**) Hershcel-Bulkley Model; (**c**) Modified Bingham Model; and (**d**) Power Law Model.

**Figure 5:**
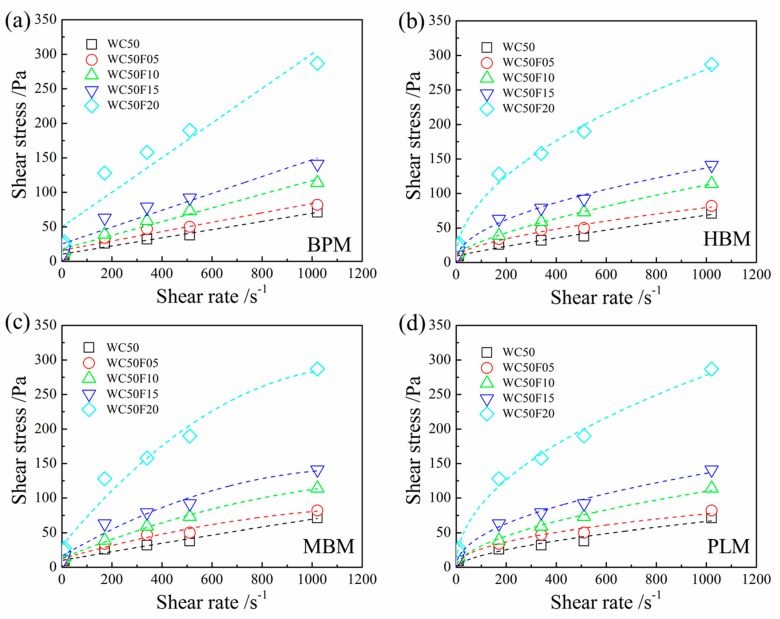
Experimental data and fitted plot of shear stress versus shear rate for CNF-OWC slurries: (**a**) Bingham Plastic Model; (**b**) Hershcel-Bulkley Model; (**c**) Modified Bingham Model; and (**d**) Power Law Model.

Figure 4 and Figure 5 show experimental data and fitted plots of shear stress versus shear rate for CNC-OWC slurries. It can be seen from Figure 4 and Figure 5 that CNCs and CNFs had quite different effects on the rheological properties of OWC paste. The shear stress of OWC slurry apparently decreased with the incorporation of CNCs at low loading (0.05 wt.%, 0.10 wt.% and 0.15 wt.%). With the incorporation of CNCs reached 0.20 wt.%, the shear stress of OWC slurry became higher than that of the control slurry. In contrast, the addition of CNFs to the OWC slurry resulted in an increase in shear stress, even at very low levels. The shear stress increased with the increase of the CNF addition amount at the same shear rate. When the content of CNFs reached 0.20 wt.%, the shear stress of OWC slurry increased greatly, much higher than that of the control slurry.

Compared with BPM, the shear stress vs shear rate curves fitted by HBM, MBM and PLM exhibited a non-linear relationship. In general, the MBM and PLM can better fit the relationship between shear stress and shear rate as shown in Figure 4 and Figure 5.

**Figure 6:**
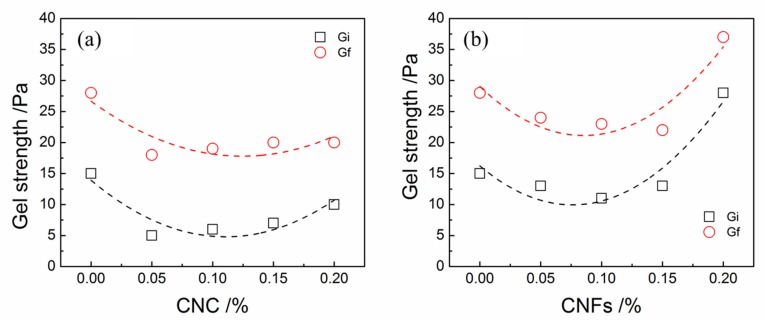
Plots of initial and final gel strengths as a function of CNP content: (**a**) CNC-OWC; (**b**) CNF-OWC.

It can be observed from Figure 6a that the additive amount of CNC increased from 0.05% to 0.2%, the initial and final gel strengths of CNC-OWC slurries gradually increased. The initial and final gel strength of CNF-OWC slurries slightly decreased first and then increased when the amount of CNC increased from 0.05% to 0.2% (Figure 6b). As for CNF-OWC slurries, when the addition quantity of CNFs increased to 0.20%, the initial and final gel strengths became higher than these of neat OWC slurries. The initial and final gel strengths of the CNC-OWC slurries were smaller than these of the neat OWC slurries with the amount of CNCs from 0.05% to 0.2%. 

Yield stress values of CNP-OWC slurries calculated by various models are shown in Table 4. Higher yield stress values of CNF-OWC composites were obtained with the increase in CNF content, regardless of the models used. For example, with the addition of 0, 0.05%, 0.10%, 0.15% and 0.20 wt.% of CNF in the OWC slurry, the corresponding yield stress values were about 9.6, 16.1, 17.99, 25.32 and 49.97 Pa obtained by BPM, respectively. Similarly, only the yield stress of OWC composites added with CNCs obtained by the BPM and MBM models had an obvious trend, increasing with the increase of the CNC addition amount ranging from 0.05% to 0.20%. The calculated yield stress values of CNC-OWC slurries were lower than those of neat OWC slurry by MBM, and only higher than those of neat OWC slurry by BPM when the CNF addition amount reached 0.20%. As indicated in Table 4, the plastic viscosity of CNP-OWC slurries increased with the increase of the addition amount of CNPs obtained by BPM and MBM. The plastic viscosity of CNF-OWC slurries was higher than that of neat OWC slurries. However, for CNC-OWC slurries, it depended on the addition quantity of CNCs. For example, with the addition of 0, 0.05%, 0.10%, 0.15%, 0.20 wt.% of CNFs in the OWC slurries and 0, 0.05%, 0.10%, 0.15%, 0.20 wt.% of CNCs in the OWC slurries, the corresponding plastic viscosities were 0.05, 0.10, 0.15, 0.21, 0.43 and 0.05, 0.04, 0.04, 0.06, 0.10 with MBM model, respectively.

The CNC showed highly pronounced XRD and FTIR peaks in comparison with these of CNFs, which showed a similar tendency for the DOC results as well. Generally, the threshold values of Zeta-potential values of stable colloids are ≥+30 or ≤−30 mV [26]. The measured Zeta-potential values of CNCs and CNFs were −39.60 and −15.93 mV, respectively. Therefore, the suspension of CNFs was easier to flocculate than the suspension of CNCs, which was consistent with the test results [5]. CNFs showed highly entangled network composed of microfibril bundles and individual microfibrils and CNCs were needle-like particles. The CNFs tended to aggregate more quickly and form network structure when being incorporated with OWC slurry than CNCs, resulting in the difference of rheological properties between CNF-OWC and CNC-OWC slurries.

As CNPs interacted with OWC particles, the rheological properties of CNP-OWC composites including the viscosity and yield stress were altered. A trend of yield stress of CNC-cement slurries was reported by Cao et al, showing that yield stress decreased first and increased with the increase of the addition of CNCs [9]. The entrapped water molecules were liberated to lower the yield stress values with the mechanism of electrosteric stabilization when the additive amount of CNCs was very low [9]. At higher loading of CNCs, the yield stress of CNC-cement composites increased as a result of CNC agglomeration, which demanded a higher force to break the coacervation. The results are in an agreement with the initial and final gel strength of CNC-OWC and CNF-OWC slurries, yield stresses of CNC-OWC slurries calculated by BPM and MBM and plastic viscosity of CNC-OWC slurries calculated by BPM, MBM and CM from this work.

**Figure 7:**
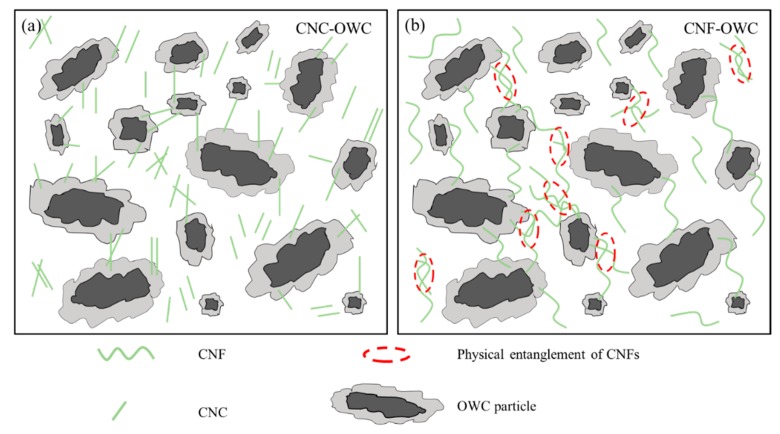
Schematic illustrations of the interaction between CNPs and OWC. (**a**) CNC-OWC; (**b**) CNF-OWC.

The influence of CNPs on the rheological properties of OWC paste mainly depends on the following two aspects. First, the CNPs suspension themselves have special rheological properties, which determine by their own morphology, surface charge and concentration. Second, the state of CNPs mixed with OWC slurry, such as the distribution of CNPs on the surface of OWC particles and in OWC paste is different. The mechanism of the influence of CNC and CNF on the rheological properties of OWC paste is schematically shown in Figure 7. In the CNP-OWC slurry, there are free-CNP and bonded/absorbed-CNP. Due to the short size of CNC, individual CNCs were more likely to be absorbed only on one OWC particle. When the concentration of CNCs was lower than the critical concentration, the CNCs acted like water reducing agent in the cement slurry. The cement slurry exhibited viscous liquid-like rheological properties, which reduced the shear stress.

However, the size of CNFs was longer, and individual OWC particles were bridged together through CNFs. In addition, the long-sized CNFs were more easily entangled to form a network structure, which caused the dispersed OWC particles to aggregate and increased the viscosity of the OWC paste. 

### 3.4. Effect of CNF Size and Mixture of CNC and CNF on Rheological Properties of OWC Slurries

**Figure 8:**
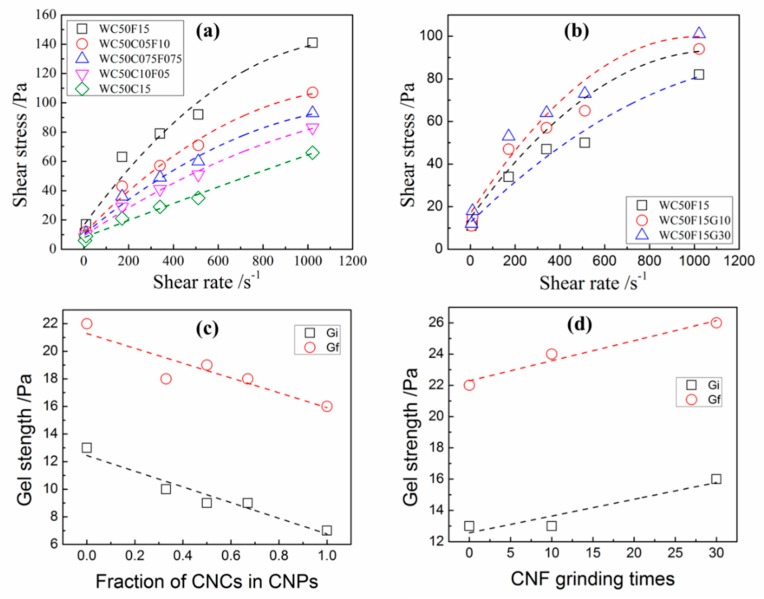
Plots of shear stress versus shear rate for CNP-OWC ((**a**) mixture of CNC and CNF and (**b**) grinding CNF-OWC, dash lines are the fitted lines using MBM) and initial and final gel strength with different addition of CNPs ((**c**) mixture of CNC and CNF and (**d**) reground CNFs).

Figure 8a,c illustrates that the shear stress and initial gel strengths of CNP-OWC slurries decreased with the increase of the proportion of CNCs in the overall CNP amount, which had a good correlation with the results shown in Figure 4 and Figure 5. It should be noted that the CNF size had a great effect on the rheological properties of CNF-OWC slurries. 

As shown in Figure 8b,d, shear stress increased with the increase of grinding times (reduced CNF size). The initial and final gel strengths followed the opposite trend. Table 4 reveals that yield stress of CNP-OWC slurries increased with the increase of the proportion of CNFs in CNP calculated by BPM and MBM, and plastic viscosity of CNP-OWC slurries showed the same trend. 

The yield stress of CNP-OWC slurries calculated by HBM did not have an obvious trend. Reduced CNF size through grinding treatment helped increase the yield stress of CNP-OWC slurries calculated by BPM and MBM, and the plastic viscosity by MBM. 

The regrinding treatment reduced the size of CNFs, but the CNF size was still much larger than that of CNCs. The overall size of CNFs, especially CNF bundles, was reduced with the increase of regrinding times, which can be seen from Figure 2c,d, and more individual CNFs formed better dispersion in the suspension. Thus, it was easier for the treated CNFs through regrinding to form an entangled network in OWC slurry and more cement particles were entangled together, which can be seen from mechanism illustration shown in Figure 7, resulting in an increase in viscosity of the OWC paste. This explains the occurrence of the phenomenon shown in Figure 8b and Table 3 and Table 4 properly that the shear stress at the same shear rate, the yield stress and plastic viscosity of CNF-OWC slurries increased with the increase of regrinding times of CNFs. Both CNFs and CNCs can promote the mechanical properties of cement composites after curing. The mechanical properties and workability of CNP-OWC composites can be balanced with the incorporation of CNPs, consisting of CNFs and CNCs with different portions. 

## 4. Conclusions

The yield stress and viscosity of neat OWC slurries had a decreasing trend with the increase of WCR. The coefficient of determination of all predicting models decreased with the increase of WCR, because of the increased fluid instability. The properties of CNPs, including rheological behaviors, surface properties and morphology, determine the rheological performance of CNP-OWC slurries. In comparison with CNC-OWC slurries, the gel strength, yield stress and viscosity of CNF-OWC slurries were higher as CNFs were more likely to form an entangled network. The initial and final gel strengths of CNC-OWC slurry were lower than these of neat OWC slurry when the CNCs addition was even up to 0.20wt%, and were consistent with the yield stress and viscosity calculated by BPM. The gel strength, yield stress and viscosity of CNP-OWC slurries increased with the reduced size through grinding treatment of CNFs and the proportion of CNFs in the mixture of CNFs and CNCs, respectively. 

The viscosity, yield stress and gel strength of the CNF-OWC slurry were higher than these of the CNC-OWC slurry due to the longer length of CNFs. The regrinding reduced the overall size of CNFs, especially large CNFs bundles and made CNFs more uniformly dispersed in the suspension, which helped form network structure and increases the viscosity of the cement paste.

## Figures and Tables

**Table 1:** Formulation of cement and cement- cellulose nanoparticles (CNP) pastes used in the study.

Formulation	Sample ID	WCR	CNP (%)
Control	WC40	0.40	0
	WC45	0.45	0
	WC50	0.50	0
	WC55	0.55	0
	WC60	0.60	0
CNC	WC50C05	0.50	0.05
	WC50C10	0.50	0.10
	WC50C15	0.50	0.15
	WC50C20	0.50	0.20
CNF	WC50F05	0.50	0.05
	WC50F10	0.50	0.10
	WC50F15	0.50	0.15
	WC50F20	0.50	0.20
	WC50F15G10	0.50	0.15
	WC50F15G30	0.50	0.15
CNC-CNF	WC50C05F10	0.50	0.15
	WC50C075F075	0.50	0.15
	WC50C10F05	0.50	0.15

Note: WC is water cement; WCR is WC ratio; C is cellulose nanocrystals (CNC); F, is CNF; G is grinding, and the added amount of CNP was based on the mass of dry oil well cement (OWC).

**Table 2:** Mathematical models for rheological properties of cement-based slurries [1,18].

Model	Main Equation
Bingham Plastic Model (BPM)	$\tau =\tau_{0}+\mu_{P}\dot{\gamma }$
Modified Bingham Model (MBM)	$\tau =\tau_{0}+\mu_{P}\dot{\gamma }+c{\dot{\gamma }}^{2}$
Power Law Model (PLM)	$\tau =k{\dot{\gamma }}^{n}$
Hershcel-Bulkley Model (HBM)	$\tau =\tau_{0}+k{\dot{\gamma }}^{n}$

Note: τ is shear stress, $\dot{\gamma }$ istrain rate, *τ*_0_ isyield stress, *μ_p_* isplastic viscosity, *c* isregression constant, *k* is consistency index, and *n* ispower law index.

**Table 3:** Rheological properties of neat OWC slurries calculated by different rheology models.

Models		Sample No.				
		WCR 0.40	WCR 0.45	WCR 0.50	WCR 0.55	WCR 0.60
BPM	*τ*_0_	18.75	10.93	9.60	10.39	8.81
	*μ*_p_	0.13	0.08	0.06	0.04	0.03
	R^2^	0.9567	0.9899	0.9660	0.8985	0.8608
MBM	*τ*_0_	10.32	9.94	10.72	13.04	10.75
	*μ*_p_	0.21	0.09	0.05	0.01	0.01
	c	−8.20 × 10^−5^	−9.59 × 10^−6^	1.09 × 10^−5^	2.58 × 10^−5^	1.89 × 10^−5^
	R^2^	0.9930	0.9912	0.9693	0.9447	0.8991
PLM	k	2.52	0.80	0.68	1.95	2.30
	n	0.58	0.68	0.66	0.44	0.38
	R^2^	0.9958	0.9721	0.9122	0.7011	0.6913
HBM	*τ*_0_	3.88	8.92	10.26	-	-
	k	1.86	0.18	0.04	-	-
	n	0.62	0.89	1.06	-	-
	R^2^	0.9968	0.9926	0.9666	-	-

**Table 4:** Yield stress and plastic viscosity of OWC slurries calculated by different rheology models.

	Sample ID	Yield Stress (Pa)			Plastic Viscosity (Pa.s)	
		BPM	MBM	HBM	BPM	MBM
Control	WC50	9.60	10.72	10.26	0.05	0.04
CNC	WC50C05	2.23	3.87	-	0.06	0.04
	WC50C10	3.90	5.28	5.48	0.06	0.06
	WC50C15	8.31	8.00	7.37	0.07	0.10
	WC50C20	12.25	9.02	6.70	0.07	0.10
CNF	WC50F05	16.10	12.54	8.65	0.10	0.15
	WC50F10	17.99	13.30	10.24	0.12	0.21
	WC50F15	25.32	16.37	4.01	0.25	0.43
	WC50F20	49.97	31.16	6.48	0.08	0.14
	WC50F15G10	21.52	14.73	3.39	0.08	0.16
	WC50F15G30	25.15	16.82	0.50	0.09	0.16
CNC-CNF	WC50C05F10	17.24	10.82	4.31	0.08	0.13
	WC50C075F075	14.92	10.10	5.69	0.07	0.10
	WC50C10F05	12.20	9.25	7.11	0.05	0.04

## References

[1] Shahriar A. (2011). Investigation on Rheology of Oil Well Cement Slurries. Ph.D. Thesis.

[2] Broni-Bediako E., Joel O.F., Ofori-Sarpong G. (2016). Oil well cement additives: A review of the common types. Oil Gas Res..

[3] Joshi R.C., Lohita R.P. (1997). Advances in Concrete Technology, 2, Fly Ash in Concrete: Production, Properties and Uses.

[4] Fu T., Moon R.J., Zavattieri P., Youngblood J., Weiss W.J. (2017). Cellulose nanomaterials as additives for cementitious materials. Cellulose-Reinforced Nanofibre Composites.

[5] Li M.C., Wu Q., Song K., Qing Y., Wu Y. (2015). Cellulose nanoparticles as modifiers for rheology and fluid loss in bentonite water-based fluids. ACS Appl. Mater. Interfaces..

[6] Nilsson J., Sargenius P. (2011). Effect of Microfibrillar Cellulose on Concrete Equivalent Mortar Fresh and Hardened Properties. Ph.D. Thesis.

[7] Moon R.J., Martini A., Nairn J., Simonsen J., Youngblood J. (2011). Cellulose nanomaterials review: Structure, properties and nanocomposites. Chem. Soc. Rev..

[8] Gandelman R., Miranda C., Teixeira K., Martins A.L., Waldmann A. (2004). On the rheological parameters governing oilwell cement slurry stability. Annu. Trans. Nord. Rheol. Soc..

[9] Flores J., Kamali M., Ghahremaninezhad A. (2017). An investigation into the properties and microstructure of cement mixtures modified with cellulose nanocrystal. Materials.

[10] Cao Y., Zavaterri P., Youngblood J., Moon R., Weiss J. (2015). The influence of cellulose nanocrystal additions on the performance of cement paste. Cem. Concr. Compos..

[11] Cao Y., Tian N., Bahr D., Zavattieri P.D., Youngblood J., Moon R.J., Weiss J. (2016). The influence of cellulose nanocrystals on the microstructure of cement paste. Cem. Concr. Compos..

[12] Ardanuy Raso M., Claramunt Blanes J., Arévalo Peces R., Parés Sabatés F., Aracri E., Vidal Lluciá T. (2012). Nanofibrillated cellulose (NFC) as a potential reinforcement for high performance cement mortar composites. BioResources.

[13] Onuaguluchi O., Panesar D.K., Sain M. (2014). Properties of nanofibre reinforced cement composites. Constr. Build. Mater..

[14] Mejdoub R., Hammi H., Suñol J.J., Khitouni M., M‘nif A., Boufi S. (2017). Nanofibrillated cellulose as nanoreinforcement in Portland cement: Thermal, mechanical and microstructural properties. J. Compos. Mater..

[15] Sun X., Wu Q., Lee S., Qing Y., Wu Y. (2016). Cellulose nanofibers as a modifier for rheology, curing and mechanical performance of oil well cement. Sci. Rep..

[16] Sun X., Wu Q., Zhang J., Qing Y., Wu Y., Lee S. (2017). Rheology, curing temperature and mechanical performance of oil well cement: Combined effect of cellulose nanofibers and graphene nano-platelets. Mater. Des..

[17] Papo A. (1988). Rheological models for cement pastes. Mater. Struct..

[18] Segal L.G.J.M.A., Creely J.J., Martin Jr A.E., Conrad C.M. (1959). An empirical method for estimating the degree of crystallinity of native cellulose using the X-ray diffractometer. Text. Res. J..

[19] Tattersall G.H., Banfill P.F. (1983). The Rheology of Fresh Concrete.

[20] Jones T.E.R., Taylor S. (1977). A mathematical model relating the flow curve of a cement paste to its water/cement ratio. Mag. Concr. Res..

[21] Lu P., Hsieh Y.L. (2010). Preparation and properties of cellulose nanocrystals: Rods, spheres, and network. Carbohydr. Polym..

[22] Sun X., Wu Q., Ren S., Lei T. (2015). Comparison of highly transparent all-cellulose nanopaper prepared using sulfuric acid and TEMPO-mediated oxidation methods. Cellulose.

[23] Vom Berg W. (1979). Influence of specific surface and concentration of solids upon the flow behaviour of cement pastes. Mag. Concr. Res..

[24] Atzeni C., Massidda L., Sanna U. (1985). Comparison between rheological models for portland cement pastes. Cem. Concr. Res..

[25] Felekoğlu B., Türkel S., Baradan B. (2007). Effect of water/cement ratio on the fresh and hardened properties of self-compacting concrete. Build. Environ..

[26] Everett D.H. (2007). Basic Principles of Colloid Science.

